# The Blitz Anesthesia Technique in Non-English Speaking Patients Undergoing Glaucoma Surgery

**DOI:** 10.5005/jp-journals-10008-1112

**Published:** 2012-08-16

**Authors:** Juliana Almodin, Parul Ichhpujani, Arun Prasad, Scott J Fudemberg, Marlene R Moster

**Affiliations:** 1 Anne and William Goldberg Glaucoma Service, Wills Eye Institute Jefferson Medical College, Philadelphia, PA, USA; 2 Anne and William Goldberg Glaucoma Service, Wills Eye Institute Jefferson Medical College, Philadelphia, PA, USA; 3 Anne and William Goldberg Glaucoma Service, Wills Eye Institute Jefferson Medical College, Philadelphia, PA, USA; 4 Anne and William Goldberg Glaucoma Service, Wills Eye Institute Jefferson Medical College, Philadelphia, PA, USA; 5 Anne and William Goldberg Glaucoma Service, Wills Eye Institute Jefferson Medical College, Philadelphia, PA, USA

**Keywords:** Blitz anesthesia, Trabeculectomy, Eye safety, Glaucoma.

## Abstract

**Aim:**

To describe a less invasive method of providing anesthesia in non-English speaking patients undergoing glaucoma surgery.

**Settings and design:**

Prospective observational study conducted in a tertiary Care Eye Institute, Wills Eye Institute, Philadelphia, PA, USA.

**Materials and methods:**

The blitz anesthesia technique was applied to 15 non-English speaking patients (Vietnamese, Mandarin, Russian and Korean) during glaucoma surgery. With input from family members, a diagram was created for each patient. The diagram consisted of a translation and phonetic guide to pronunciation of common words or phrases in the patient’s native language that might be used by the surgical team during the operation.

**Results:**

The blitz anesthesia technique worked well to provide patient comfort during the procedures. All patients reported adequate pain control and described their experience as comfortable. Additionally, patients reported feeling reassured that they were able to understand basic information from the surgical team during their case. This technique decreased patient anxiety prior to and during the surgical procedure.

**Conclusion:**

Blitz anesthesia provided adequate pain control with no complications.

**Key message:**

Blitz anesthesia with a phonetic language diagram, a less invasive technique of providing anesthesia in non-English speaking patients undergoing glaucoma surgery.

**How to cite this article:**

Almodin J, Ichhpujani P, Prasad A, Fudemberg SJ, Moster MR. The Blitz Anesthesia Technique in Non-English Speaking Patients Undergoing Glaucoma Surgery. J Current Glau Prac 2012;6(2):91-93.

## INTRODUCTION

In a multicultural country, like United States, despite an attempt toward acculturation, physicians have to encounter non-English speaking people in various clinical settings.^1^ Physicians may feel inadequacy due to communication barriers while operating on such patients. These patients are more apprehensive during preoperative and peroperative period.^2^

Numerous techniques exist for administering anesthesia for ophthalmic surgery. These include retrobulbar, peribul-bar, parabulbar (subconjunctival and subtenon’s), topical and general anesthesia. Appropriate reasons for each method and their associated strengths and weakness are well know to ophthalmologists.^3^ The blitz anesthesia technique which includes supplementing the topical anesthesia (2% xylocaine jelly), along with intracameral lidocaine and anterior sub-Tenon’s anesthesia, has proven to be beneficial in terms of patient comfort and anesthesia complications.^4^

This report describes blitz anesthesia with a phonetic language diagram, a less invasive technique of providing anesthesia in non-English speaking patients undergoing glaucoma surgery. Phonetic language diagram can be useful especially in the surgical centers, where bilingual professionals or interpreters are not available.^5^ By applying the blitz technique to these patients and facilitating communication, we feel that a faster recovery with fewer anesthetic risks can be accomplished.

## MATERIALS AND METHODS

In this prospective observational study, 15 non-English speaking patients (Vietnamese, Mandarin, Russian and Korean) undergoing glaucoma surgery at Wills Eye Institute were recruited. The study conformed to the tenets of the declaration of Helsinki. All patients signed an informed consent form approved by the institutional review board of Wills Eye Institute. Inclusion criteria included patients who needed glaucoma surgery without general anesthesia and were unable to communicate in English. Exclusion criteria were significant barriers to communication besides language (deafness or cognitive impairment) and reported allergy to lidocaine.

Main outcome measures included patient comfort, ability of patient to follow intraoperative commands and successful completion of glaucoma surgery. With input from family members, a diagram was created for each patient. The diagram consisted of words or phrases in the patient’s native language that might commonly be used during surgery with a translation and phonetic guide to pronunciation for the surgical team. All patients received Blitz anesthesia during glaucoma surgery.

No patients received sedatives before entering the operating room. All patients received a standardized intravenous sedative consisting of midazolam 1 mg and fentanyl 50 |ug. Three of the 15 patients (20%) required propofol 30 to 50 mg as they felt discomfort. A paracentesis was performed and a small volume of aqueous was allowed to drain from the eye. One tenth of a cc of 1% nonpreserved lidocaine was injected into the anterior chamber with a 27 gauge cannula. For fornix-based filters, a 4 to 5 mm limbal incision was created. Approximately, 0.5 cc of1% nonpreserved lidocaine was injected in the sub-Tenon’s space anterior to the muscle insertions with the tip of the cannula pointing posteriorly to ensure broad coverage. For limbal-based filters, following the intracameral injection, a 30 gauge needle on the same syringe was inserted through the conjunctiva and Tenon’s capsule 10 mm posterior to the limbus in a superior quadrant, and approximately 0.5 cc of 1% nonpreserved lidocaine was injected. Then either a 4-0 silk superior rectus suture or an 8-0 limbal traction suture was placed in preparation for the filtration procedure. Prior to conjunctival closure for both fornix and limbal based flaps, approximately 0.5 cc of nonpreserved lidocaine was injected into the incision of the conjunctiva and Tenon’s. The wound was then closed with 8-0 vicryl for limbus based flaps and 10-0 nylon for fornix-based procedures. A plastic shield was placed on the operated eye after conclusion of the procedure.

## RESULTS

During the procedure, the surgeons communicated with the each patient by reading the translations from a poster hanging close to the operative field made with the help of the patients’ families. The surgical team felt comfortable in speaking the foreign language because all terms were spelled phonetically in English allowing for accurate pronunciation (Figs 1A and B).

The translations included terms, such as look up, down, left, right as well as statements, such as ‘do not move’, ‘look at the light’ and ‘any pain?’ Most importantly, all of the patients responded appropriately to intraoperative commands allowing the surgery to proceed safely.

**Fig. 1A:**
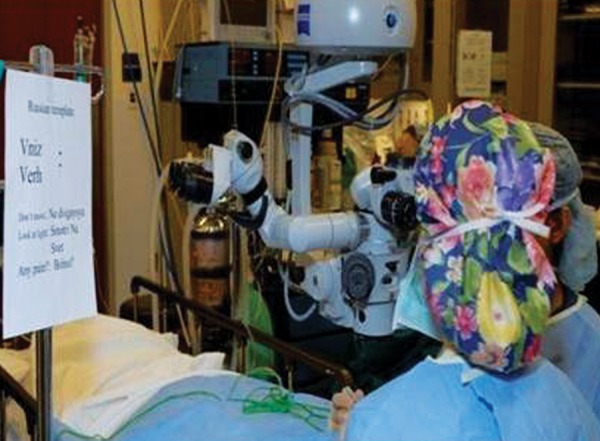
Russian template showing common phrases spelt phonetically in English

**Fig. 1B:**
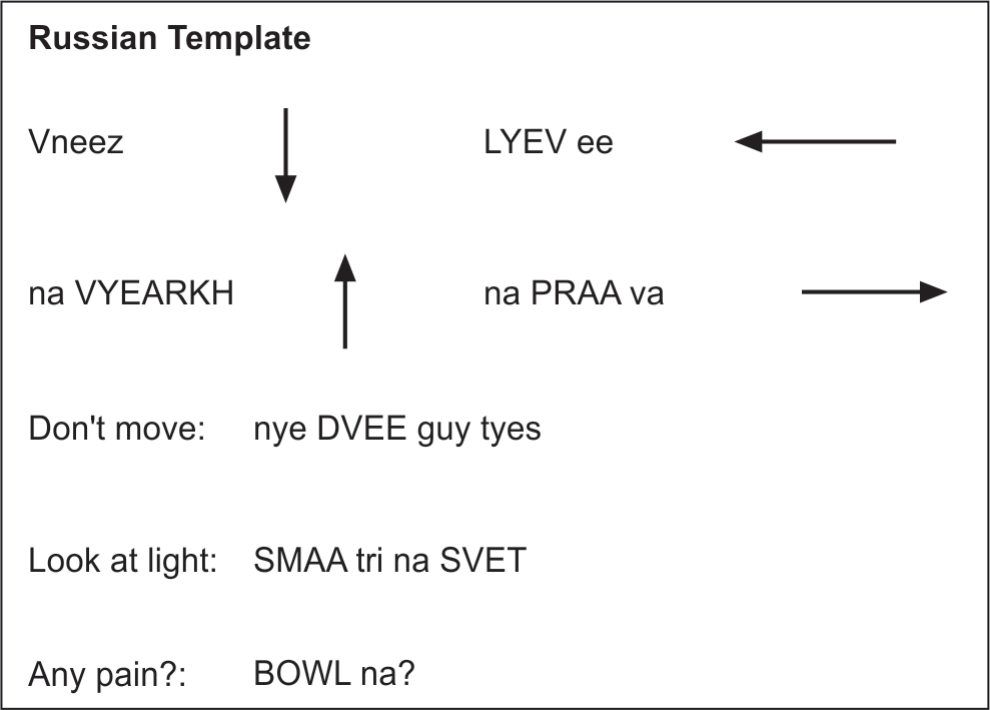
Phonetic line diagram-assisted communication during surgery

At the conclusion of the procedure, all patients were asked a series of questions as regards to their surgical experience. They were asked if their pain was adequately controlled during the procedure, if they felt comfortable in their ability to communicate with the surgeons, and finally if they were able to understand the surgeons’ requests and questions. In response to these questions, all 15 patients (100%) reported that they felt that their pain was satisfactorily managed during the procedure, and they were able to both understand the surgical commands as well as relay their own feelings. Also, all patients indicated that knowing that direct communication with the surgeons would be possible during the case was comforting both prior to and during the surgery.

Lastly, by employing the blitz technique all of the anesthetic risks associated with retrobulbar, peribulbar and general anesthesia were avoided. No complications from anesthesia were noted in any of these patients.

## DISCUSSION

Retrobulbar and peribulbar anesthesia are by far the most commonly used forms of local anesthesia in glaucoma surgeries. However, they are not without their risks which can be both sight and life-threatening.^3^ Alternatively, Kansal et al have reported that employing the blitz technique minimizes any such anesthesia risk, while maintaining an adequate level of pain control.^4^ Hence, blitz technique allows for both patient comfort as well as a decrease in the amount of unwanted eye movements creating a safe surgical environment for surgeons’ ease.

Multiple studies have shown that alternatives to the classical retrobulbar and peribulbar anesthesia, such as subconjunctival^6,7^ and intracameral^7,8^ techniques, have been equally effective in providing a comfortable peropera-tive experience for the patient. In such settings, the patient maintains the ability to perform eye movements and, thus, it is imperative that an adequate level of communication is possible between the surgeon and the patient. As one may imagine, a serious hindrance to this procedure would be a non-English speaking patient with whom the surgeon is unable to communicate with. The patients would not be able to follow important instructions, such as ‘look up’ or ‘do not move’, if the surgeon did not speak their language. Our study attempted to find a way to overcome this cultural barrier and make the blitz technique a reasonable or even a first line choice in non-English speaking patients. By learning how to pronounce important instructions in the patient’s native tongue, our surgeons have demonstrated that an adequate level of understanding can be established between the two parties. Such an understanding has proven to be beneficial for the performance of the surgical procedure and also for the patient’s psychological state. All patients stated that they were less anxious knowing that the surgeon would be able to communicate with them during the glaucoma operation.

## CONCLUSION

Blitz anesthesia with a phonetic language diagram is a novel tool that can foster quality communication with patients especially during intraoperative period.
